# The Intrapopulation Genetic Diversity of RNA Virus May Influence the Sensitivity of Chlorine Disinfection

**DOI:** 10.3389/fmicb.2022.839513

**Published:** 2022-05-20

**Authors:** Syun-suke Kadoya, Syun-ichi Urayama, Takuro Nunoura, Miho Hirai, Yoshihiro Takaki, Masaaki Kitajima, Toyoko Nakagomi, Osamu Nakagomi, Satoshi Okabe, Osamu Nishimura, Daisuke Sano

**Affiliations:** ^1^Department of Civil and Environmental Engineering, Graduate School of Engineering, Tohoku University, Sendai, Japan; ^2^Department of Urban Engineering, The University of Tokyo, Tokyo, Japan; ^3^Graduate School of Life and Environmental Sciences, University of Tsukuba, Tsukuba, Japan; ^4^Research Center for Bioscience and Nanoscience, Japan Agency for Marine-Earth Science and Technology, Yokosuka, Japan; ^5^Super-Cutting-Edge Grand and Advanced Research Program, Japan Agency for Marine-Earth Science and Technology, Yokosuka, Japan; ^6^Division of Environmental Engineering, Faculty of Engineering, Hokkaido University, Sapporo, Japan; ^7^Department of Molecular Microbiology and Immunology, Nagasaki University, Nagasaki, Japan; ^8^Department of Frontier Sciences for Advanced Environment, Graduate School of Environmental Studies, Tohoku University, Sendai, Japan

**Keywords:** RNA virus, disinfection, genetic diversity, quasispecies, evolutionary experiment

## Abstract

RNA virus populations are not clonal; rather, they comprise a mutant swarm in which sequences are slightly different from the master sequence. Genetic diversity within a population (intrapopulation genetic diversity) is critical for RNA viruses to survive under environmental stresses. Disinfection has become an important practice in the control of pathogenic viruses; however, the impact of intrapopulation genetic diversity on the sensitivity of disinfection, defined as –log_10_ (postdisinfected infectious titer/predisinfected titer), has not been elucidated. In this study, we serially passaged populations of rhesus rotavirus. We demonstrated that populations with reduced chlorine sensitivity emerged at random and independently of chlorine exposure. Sequencing analysis revealed that compared with sensitive populations, less-sensitive ones had higher non-synonymous genetic diversity of the outer capsid protein gene, suggesting that changes in the amino acid sequences of the outer capsid protein were the main factors influencing chlorine sensitivity. No common mutations were found among less-sensitive populations, indicating that rather than specific mutations, the diversity of the outer capsid protein itself was associated with the disinfection sensitivity and that the disinfection sensitivity changed stochastically. Simulation results suggest that the disinfection sensitivity of a genetically diverse population is destabilized if cooperative viral clusters including multiple sequences are formed. These results advocate that any prevention measures leading to low intrapopulation genetic diversity are important to prevent the spread and evolution of pathogenic RNA viruses in society.

## Introduction

Although RNA virus populations are not clonal, they comprise mutant swarms that have different genomic sequences from the master sequence and are, therefore, called quasispecies (Domingo et al., 1996; Domingo and Holland, 1997). Mutants in these swarms have one or more point mutations and indels on their genome due to high mutation rates, and this allows the viruses to establish a genetically diverse population (Sanjuán et al., 2010; Duffy, 2018). The genetic diversity of an RNA virus is crucial in its adaptation to environmental changes, and even rare mutants within virus populations significantly affect the population phenotype (Perales et al., 2012). For example, the rabies virus is known to have adapted to new environments without the replacement of its master sequence (Kissi et al., 1999). The replicative ability of a West Nile virus population became high with increases in its genetic diversity (Ciota et al., 2007). Various minor mutants in a coxsackievirus population helped the dominant strain adapt to a change in cell tropism (Bordería et al., 2015).

Increases in genetic diversity are usually restricted by some forces: natural selection and genetic drift owing to a bottleneck (Domingo, 2016). Advantageous mutations can be propagated by natural selection in one population until they become disadvantageous in other environments, and the population structure of the next generation is randomly determined when a bottleneck occurs (Domingo, 2016). The within-host genetic diversity of the dengue virus is reportedly influenced by both genetic drift and selection (Lequime et al., 2016). Repeatedly, strong bottlenecks reduced the capacity of the foot-and-mouth disease virus to produce infectious progenies but did not cause its extinction (Lázaro et al., 2003). Moreover, bottlenecks enable one of the plant RNA viruses to fix adaptive subpopulations (or mutants) within a population during cell-to-cell transmission (Miyashita and Kishino, 2010). Thus, both selection and bottlenecks alter the structure and phenotypes of virus populations.

Enteric viruses such as rotavirus and norovirus are RNA viruses and spread from human fecal matter to various environments. Wastewater, which contains pathogenic RNA viruses, is often reused for irrigation after disinfection treatments (World Health Organization, 2015) or is released into water bodies. At the household level, virus particles from human feces or vomiting- and sneezing-related aerosols can adhere to various environmental surfaces (Abad et al., 1994). To reduce the risk of infection from RNA viruses, disinfection has been commonly used as a preventive measure. Appropriate disinfection of treated wastewater and environmental surfaces, as well as proper food processing, are important to prevent the spread of viruses in human society. However, a previous study reported that disinfection altered the population structure of a gastroenteritis-causing virus (Rachmadi et al., 2018). Thus, it is important to understand how an RNA virus quasispecies responds to disinfection to help us develop a novel strategy that can prevent RNA viruses from spreading to humans; for example, we must determine the appropriate disinfection level that sufficiently inactivates virus populations with reduced disinfection sensitivity.

In this study, we used populations of rotavirus as a representative of pathogenic RNA viruses and assessed how the virus populations altered their sensitivity to disinfection. Rotavirus is a double-stranded RNA (dsRNA) virus with 11 genome segment coding structural proteins (VP1, 2, 3, 4, 6, and 7) and non-structural proteins (NSP1, 2, 3, 4, and 5/6), and its virion is composed of three capsid layers (VP7: outer capsid protein, VP6: inner capsid, and VP2: core protein), a spike protein (VP4), and an enzymatic complex of VP1 and VP3. We identified the mechanism underlying the change in disinfection sensitivity. We demonstrated that the disinfection sensitivity of rotavirus randomly changed and that its change was associated with the diversity of non-synonymous mutations (non-synonymous genetic diversity) of the outer capsid protein gene (VP7). There were no specific mutations in less-sensitive populations, indicating that the genetic diversity may be correlated with the disinfection sensitivity of the rotavirus population. Viral phenotypes such as infectious titer, cell-binding ability, specific growth rate, and lag period were not different between the less-sensitive and sensitive populations. Since non-synonymous mutations on the protein surfaces can change various physical properties such as electrostatic potential and hydrophobic interaction, we hypothesized that virions with different sequences tended to gather each other (cooperative virus cluster) within a genetically diverse population, resulting in the reduction of disinfection sensitivity. Based on this hypothesis, we performed an evolutionary simulation and observed that the disinfection sensitivity was likely determined by cooperation capable of maintaining the genetic diversity and by the stochasticity of successful transmission of cooperative virus clusters to the next generation.

## Materials and Methods

### Cell and Virus

**FIGURE 1:**
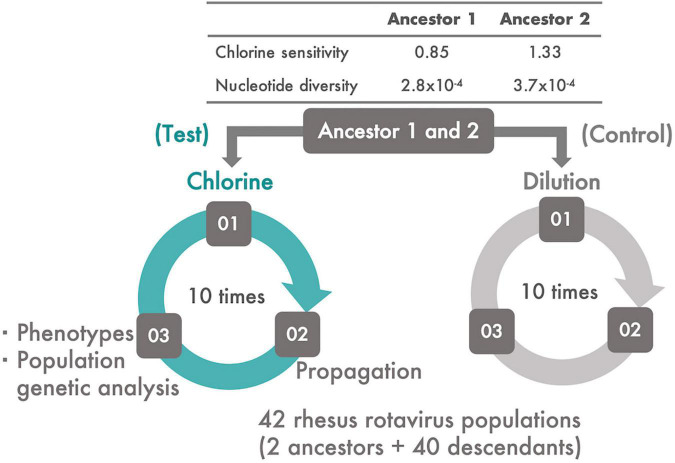
The experimental system. Two ancestral populations of RRV, which had different chlorine and genetic diversities, were serially passaged 10 times using MA104 cells. From each ancestral population, two lines of serial passages were established; one was subjected to disinfection with chlorine (Test) and the other to dilution (Control). During the serial passages, viral phenotypes (infectious titer, cell-binding ability, specific growth rate, and lag time) and chlorine sensitivity of all the populations were evaluated. In addition, the frequency of the transition of single nucleotide polymorphisms (SNPs) and synonymous/non-synonymous nucleotide diversity was estimated.

MA104 cells (ATCC:CRL-2378.1™) were cultured in Eagle’s minimal essential medium (MEM) containing 10% fetal bovine serum, 2 mM L-glutamine, 1% penicillin–streptomycin (GIBCO by Life Technology), and 1.125 g/L sodium bicarbonate (Wako Pure Chemical Industries, Ltd., Osaka, Japan) in a T75 flask. The average cell numbers were approximately 5.0 × 10^6^ cells/flask. The original rhesus rotavirus (RRV) population was passaged thrice using the MA104 cell line. Trypsin from porcine pancreas type IX-S (Sigma-Aldrich, Burlington, MA, United States) at a final concentration of 40 μg/ml was added to a 1 ml RRV suspension to facilitate viral entry into the MA104 cells; the incubation conditions were 37°C for 30 min under 5% CO_2_. After incubation and discarding of the cell culture medium in a flask in which MA104 cells were confluent, the RRV suspension was inoculated onto the cells at a multiplicity of infection (MOI) of 0.1 and incubated at 37°C for a further 60 min under 5% CO_2_ to allow the viruses to bind the cell receptor. Then, 32 ml of serum-free Eagle’s MEM was added to the infected culture and maintained for 2 or 3 days. At the time of harvest, the flask was frozen and thawed thrice to recover not only the viral particles released from the cells but also those that remained within the cells. The resulting suspension was centrifuged at 12,600 *× g* for 10 min at 4°C to remove cell debris. The same procedure was performed in duplicate on the original RRV population to generate two ancestral populations for serial passage experiments. The population structure, nucleotide diversity, and sensitivity of these ancestral populations to chlorine were different from each other (Figure 1 and Supplementary Figure 2).

### Disinfection Condition

**FIGURE 2:**
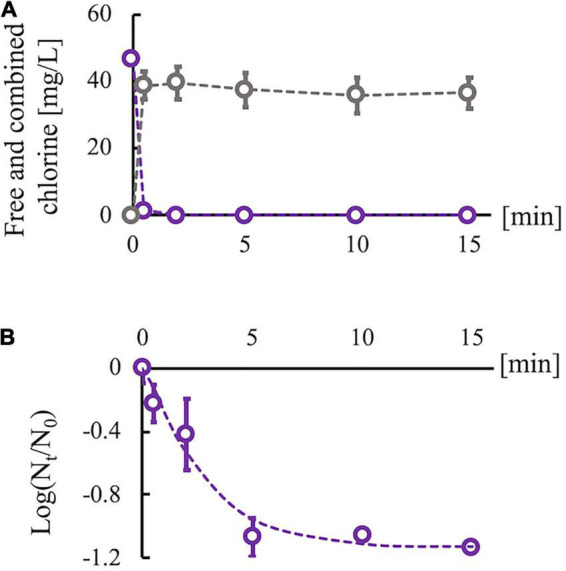
Changes in chlorine concentration and infectious titer of rhesus rotavirus (RRV). **(A)** Changes of free (purple) and combined chlorine (gray) concentrations under the existence of organic matter (cell culture medium). **(B)** Inactivation curve of RRV with 45 mg/L of initial free chlorine concentration, which is approximated by the efficiency Hom factor model (dashed line). Plots are observed data, and error bars show the standard deviation (*N* = 3) in all experiments. The log_10_ reduction value reached 1 log reduction at 10 min and became stable.

Sodium hypochlorite (Wako Pure Chemical Industries, Ltd., Osaka, Japan) was diluted with Dulbecco’s phosphate-buffered saline (PBS) (–) (Nissui Pharmaceutical Co., Ltd., Tokyo, Japan) to adjust the concentration of free chlorine. The free chlorine concentration was measured using the *N*,*N*-diethyl-*p*-phenylenediamine method. An RRV suspension was disinfected using chlorine (room temperature, pH 7.6) without purification because drinking water, wastewater, and environmental surfaces polluted by feces are usually associated with disinfectant-consuming organic matter. Then, 0.5 ml of the RRV suspension (10^6^ PFU/ml) was added to 4.5 ml of chlorine solution (initial free chlorine concentration, 45 mg/L). The mixed suspension was sampled after 0, 0.5, 2, 5, 10, and 15 min, and the residual chlorine in the sampled suspension was neutralized using 1% sodium thiosulfate of which concentration properly neutralizing the residual chlorine was determined on the basis of the reaction’ stoichiometry of chlorine and sodium thiosulfate. The concentration of sodium thiosulfate used for the neutralization did not prevent cell growth and virus propagation. We also measured the total (free and combined) chlorine concentration using the *N*,*N*-diethyl-*p*-phenylenediamine method because combined chlorine is easily generated *via* the reaction of free chlorine with organic matter in virus suspensions and can inactivate the viruses. Due to the consumption of free chlorine species (HClO and ClO^–^) by organic components, the free chlorine concentration decreased immediately, but some residual free chlorine remained (0.06–0.08 mg/L) (Figure 2A). In contrast, the combined chlorine concentration increased at 0.5 min and remained stable thereafter. The infectivity of the predisinfected and postdisinfected RRV populations was confirmed using plaque assay (described in the “Serial passage experiments” section). A time-course change of the log reduction value (LRV) {ratios of infectious titer [PFU/ml] at contact time *N*_*t*_ to that before disinfection *N*_0_ “Log_10_ (*N*_*t*_/*N*_0_)”} was fit to the efficiency of the Hom factor model to yield an inactivation curve (Haas and Joffe, 1994). The inactivation curve achieved approximately 1.0 LRV (equal to 90% inactivation) at 5 min and remained constant even after 10 min (Figure 2B). In addition, chlorine decay was stable at 10 min (Figure 2A); therefore, the contact time for the serial passage experiments was set to 10 min. Taken together, this chlorine dosing condition (initial free chlorine concentration: 45 mg/L and contact time: 10 min) was adopted for all serial passage experiments. All experiments to determine the disinfection conditions were conducted thrice (*N* = 3).

### Serial Passage Experiments

Each serial passage experiment had three steps: disinfection (or dilution) of an RRV population, propagation of the disinfected (or just diluted) population using the MA104 cell line, and evaluation of the phenotypes [chlorine sensitivity, infectious titer, cell binding ability, specific growth rate, and lag period of each generated descendant (Figure 1)]. In this study, the two RRV ancestral populations were serially passaged 10 times with a chlorine disinfection treatment (Test). Descendant populations derived from the two ancestral populations were defined as the first and second groups, respectively. The two ancestral populations were also serially passaged using a 100-fold dilution of Dulbecco’s PBS because it is compatible with the chlorine treatment (Control). A total of 40 descendant populations were generated *via* the serial passaging of the two initial populations with/without disinfection.

### Infectious Titer and Chlorine Sensitivity

Infectious titer and chlorine sensitivity were determined by performing the plaque assay. In line with our previous report (Kadoya and Sano, 2019), serially diluted virus suspensions were treated with trypsin obtained from the porcine pancreas. Then, the RRV suspensions were inoculated onto confluent MA104 cells in 6-well plates; the plates were incubated at 37°C for 90 min under 5% CO_2_. After the cells were washed twice with Dulbecco’s PBS (–), 2.5% agar mixed with the same volume of phenol red free-MEM (2% fetal bovine serum, 2% penicillin–streptomycin, 4 mM L-glutamine, 2.25 g/L NaHCO_3_, and 40 μg/ml trypsin from the porcine pancreas) was overlayed, and the plate was incubated for 2 days. MA104 cells were stained with 0.015% neutral red for 3 h at 37°C for 90 min under 5% CO_2_, and then, neutral red was removed. After 1 or 2 days, the number of plaques was counted. Chlorine sensitivity was taken as LRV (–log_10_ [postdisinfected/predisinfected infectious titer]) and measured at the same disinfection conditions as that in the serial passage experiments. The infectious titer and chlorine sensitivity were measured thrice for all experiments using the plaque assay.

### Cell-Binding Efficiency

A cell-binding assay was conducted thrice following the steps outlined in our previous report (Kadoya and Sano, 2019). Confluent MA104 cells were washed twice with tris-buffered saline. Then, 100 μl of the suspension containing chlorine-inactivated RRV was inoculated into each well (MOI: 1.0) and incubated at 4°C for 1 h with agitation every 15 min. Three wells were used for each virus population. Then, the cells were washed thrice with tris-buffered saline, and 140 μl of Dulbecco’s PBS and 560 μl of RNA extraction buffer (QIAamp Viral RNA Mini Kit: Qiagen, Venlo, Netherland) were added. The total RNA was extracted according to the manufacturer’s instructions, incubated at 95°C for 5 min, and placed on ice for 2 min for the dsRNA to denature to single-stranded (ss) RNA. The ssRNA was reverse transcribed to cDNA using the PrimeScript RT Reagent Kit (Perfect Real Time; Takara Bio Inc., Kusatsu, Japan) following the manufacturer’s instructions. Then, qPCR was performed using Premix ExTaq (Perfect Real Time), a TaqMan probe, and an Applied Biosystems 7500 Real-Time PCR System (Thermo Fisher Scientific, Waltham, MA, United States). The sequences of forward and reverse primers and the probe that targeted the NSP3 gene segment were taken from a previous study (Pang et al., 2004). The qPCR cycling conditions were as follows: 1 cycle of an initial denaturation step at 95°C for 5 min followed by 45 cycles at 94°C for 20 s and 60°C for 1 min. Finally, one cycle of 72°C for 5 min was conducted as the extension step. The ratio of the genome copies *G*_*t*_ of the virus particles attached to cell surfaces to those inoculated to cells *G*_0_ was calculated as *G_*t*_/G_0_*.

### Growth Characteristics

A modified form of the Gompertz model was used to generate viral growth curves under substrate limitations (e.g., the number of cells), *via* which the quantitative parameters of viral growth were obtained (e.g., specific growth rate and lag period) (Zwietering et al., 1990). As in the previous study by Kadoya and Sano (2019), a disinfected RRV population was inoculated in a T25 flask (MOI: 0.01). The supernatants in the flask were obtained after 0, 6, 12, 18, 24, and 36 h, and the infectious titers were then measured using the plaque assay. A series of these experiments were conducted for 10 less-sensitive and 10 sensitive populations and replicated thrice. To estimate the specific growth rate μ and the lag time λ (the time at which the virus progeny begins to generate), the modified Gompertz model was applied to the dataset:


(1) $$L\,o\,g_{10}\,\left(N_{t}/N_{0}\right)=A\,e\,x\,p\,\left[1-e\,x\,p\,\left(\mu \,e\,\left(\lambda -t\right)/A\right)\right]$$

where *N*_0_ and *N*_*t*_ are the virus titer values (PFU/ml) at 0- and *t*-h after inoculation, *A* is the asymptotic value [*log_10_(N_∞_/N_0_)*], μ is the specific growth rate [hour^–1^], λ is the lag time [hour], and *e* is the Napier’s constant. These parameters (μ, λ, and *A*) were determined using the ordinary least-squares method.

### Fragmented and Primer-Ligated dsRNA Sequencing

As compared with conventional RNA-seq, the fragmented and primer-ligated dsRNA sequencing (FLDS) method for dsRNA sequencing enables higher sequence coverage of the entire genomic region that includes terminal sequences (Urayama et al., 2016, 2018). In brief, total nucleic acids were extracted from the infected cell culture supernatants using phenol–chloroform–isoamyl alcohol (25:24:1 ratio, pH5) (Nacalai tesque, Kyoto, Japan) and then fractionated into pure dsRNA using cellulose D (Advantec, Tokyo, Japan). Full-length dsRNA was fragmented into the 1500-bp dsRNA by sonication at 4°C for 35 s using Covaris S220 (Woburn, MA, United States); the fragments were then ligated with PC3–T7 loop primer (5′-p-GGA TCC CGG GAA TTC GGT AAT ACG ACT CAC TAT ATT TTT ATA GTG AGT CGT ATT A-OH-3′) for cDNA synthesis (SMARTer Race 5′/3′ Kit, Takara Bio, Shiga, Japan) and amplification (KOD-Plus-Neo, Toyobo, Osaka, Japan). The amplified cDNA was again fragmented into 400-bp DNA sequences using sonication at 4°C for 55 s with Covaris S400 and purified using AMPure XP (Beckman Coulter, Brea, CA, United States). An adapter sequence (SeqCap Adapter Kit A, Roche Diagnostics, Basel, Switzerland) was ligated to the cDNA using the KAPA Hyper Prep Kit (KAPA Biosystems, Woburn, MA, United States). To adjust the concentration, the cDNA library was amplified using KAPA HiFi Hot Start Ready Mix (KAPA Biosystems, Woburn, MA, United States) after purification with AMPure XP. Finally, we measured the concentration of the cDNA library using the Qubit 4 Fluorometer (Thermo Fisher Scientific, Waltham, MA, United States) and Agilent 2100 Bioanalyzer (Agilent Technology, Santa Clara, CA, United States). The subsequent 300-bp paired-end sequences were determined using Illumina MiSeq (Illumina Inc., San Diego, CA, United States).

### Sequencing Data Processing

MiSeq sequencing reads were subjected to adaptor clipping and quality trimming using Trimmomatic version 0.3344. PhiX sequences—-which are well-characterized, small genome sequences used as sequencing controls to monitor the sequencing quality—-and experimentally contaminated sequences were eliminated using the mapping tool Bowtie2 version 2.2.9 (Langmead and Salzberg, 2012). The primer sequences used for cDNA synthesis and amplification were trimmed using Cutadapt version 1.15 (Martin, 2011). Low-quality reads and PCR duplicates were detected and eliminated using PRINSEQ version 0.20 (Schmieder and Edwards, 2011). The processed reads were mapped to the reference sequences of 11 rotavirus gene segments (accession numbers: EF583006.1, EF583007.1, EF583008.1, M18736.1, HQ665465.1, EU636929.1, AY065842.1, EU636931.1, M21650.1, EU636933.1, and EU636934.1), and the consensus sequence was determined using CLC Genomics Workbench version 11 (CLC Bio, Aarhus, Denmark). Previously determined consensus sequences were used as reference sequences for each RRV genome segment (Kadoya et al., 2020). The sequence reads were mapped to the reference sequence (“Map reads to reference”) and then realigned locally to modify the gap generated in the mapping to reference sequences (“Local realignments”). Sequence coverage was obtained by applying BAM files containing information on the mapped and locally realigned reads to SAMtools version 1.8 (Li et al., 2009). The frequency of single nucleotide polymorphisms (SNPs) was estimated *via* “Low-frequency variant detection,” which is a function of the CLC Genomics Workbench and detects SNPs on the basis of a Bayesian model (both prior and posterior probabilities are estimated by an expectation-maximization algorithm). The threshold value of sequencing error frequency was set to 1.0% to avoid the detection of false-positive variants, according to the results of previous studies (Sobel Leonard et al., 2016; Wasik et al., 2019). Moreover, SNPs that were not covered by less than 10 reads were filtered out. To include information on substitution types (non-synonymous or synonymous) to the observed SNPs, the “Amino acid changes” function of the CLC Genomics Workbench was used.

### Estimation of Nucleotide Diversity

SNPGenie software, developed by Nelson et al. (2015) based on the Nei–Gojobori method (Nei and Gojobori, 1986), was used to calculate the nucleotide diversity, which has been expressed below:


(2) $$\pi =\sum_{i=1}^{s}D_{i}/L$$

(3) $$D_{i}=\frac{A_{i}\,C_{i}+A_{i}\,G_{i}+A_{i}\,T_{i}+C_{i}\,G_{i}+C_{i}\,T_{i}+G_{i}\,T_{i}}{\left(m_{i}^{2}-m_{i}\right)/2}$$

where *s* is the polymorphic site; *D*_*i*_ is the proportion of nucleotide differences at the *i*^th^ site; *L* is the length of the sequence; *A*_*i*_, *C*_*i*_, *G*_*i*_, and *T*_*i*_ are the counts for four bases at the *i*^th^ site, and *m*_*i*_ is the sequence coverage at the *i^th^* site. Synonymous and non-synonymous nucleotide diversities were calculated in a manner similar to that for nucleotide diversity, but *D*_*i*_ was modified according to the pairs of synonymous or non-synonymous substitutions.

### Exploration of Common Mutations Among Less-Sensitive Populations

The linear support vector machine (SVM) is a classification algorithm that explores a boundary line to classify samples into two distinct groups (Pedregosa et al., 2011). In this study, a boundary line was expressed as a linear function using the frequency of non-synonymous SNPs on the VP7 genome segment:


(4) $$f\,\left(x\right)=\omega^{T}\,\text{x}+b$$

where **ω** is the vector for coefficients (*ω_1_*, *ω_2_*,…, *ω_*n*_*) of ***x*** that is also a vector of the frequency of non-synonymous SNPs (SNP_1_, SNP_2_,…, SNP_n_); *b* is an intercept; and *n* is the number of SNPs on the VP7 genome segment (*N* = 62). Then, we denoted another function for binary classification *g*(*t*), which is 1 if *f*(*x*) ≧ 0; otherwise, it is –1. To determine *f*(*x*), the algorithm seeks data points belonging to different classes nearest to the boundary line. The **ω** and *b* are then calculated to maximize the distance of the boundary line from support vectors. Classification into less-sensitive groups could have been determined by only a few SNPs; thus, we introduced linear SVM into the L1 regularization term. L1 regularization mathematically cuts the coefficient of non-essential variables, which is estimated as zero. In contrast, L2 regularization employs all variables that are shrunk as small as possible. The total dataset was separated into the train (80%) and test datasets (20%), and the boundary line was estimated using only the train dataset. The test dataset was then predicted by the estimated model, and the prediction performance was evaluated on the basis of the accuracy defined below:


(5) $$A\,c\,c\,u\,r\,a\,c\,y=\frac{T\,P+T\,N}{T\,P+T\,N+F\,P+F\,N}$$

where *TP*, *TN*, *FP*, and *FN* are the numbers of true positives, true negatives, false positives, and false negatives, respectively. Linear SVM was conducted using the scikit-learn library in Python version 3.7^1^.

### Evolutionary Simulation

Simultaneous ordinary differential equations, which have been originally established for simulating the population dynamics of the human immune deficiency virus under immune pressures specific to each strain, were applied (Nowak, 2006) and modified to express the effects of disinfection on virus population sizes. The first equation describes the log_10_ population size of subpopulation *i* (*v*_*i*_) as follows:


(6) $$\dot{v_{i}}=r_{i}\,v_{i}-p\,x_{i}\,v_{i}$$

where *r*_*i*_ is the overall growth ability and *p* is the coefficient of a removal term associated with practical inactivation efficiency (*px_*i*_v_*i*_*). The response of subpopulation *i* to chlorine (*x*_*i*_) is proportional to the log_10_ virus population size (*cv*_*i*_) but is restrained by the subpopulation-specific sensitivity (*b_*i*_x_*i*_*), which is expressed as follows:


(7) $$\dot{x_{i}}=c\,v_{i}-b_{i}\,x_{i}$$

If subpopulation *i* positively interacts with others, the terms of the subpopulation-specific sensitivity of other subpopulations are added to the equation of *x*_*i*_:


(8) $$\dot{x_{i}}=c\,v_{i}-\left(b_{i}\,x_{i}+b_{i+1}\,x_{i+1}+\cdots \right)$$

We further assumed that bottlenecks exerted on this viral population and excluded some cooperative subpopulations, which resulted in random changes in the number of positive interactions:


(9) $$\dot{x_{i}}=c\,v_{i}-\left(b_{i}\,x_{i}\,\theta_{i}+b_{i+1}\,x_{i+1}\,\theta_{i+1}+\cdots \right)$$

The parameter θ followed binomial distribution, and it took 0 or 1 to express the bottleneck. If *θ_*i* + 1_* is 0, subpopulation *i* loses the interaction with subpopulation *i + 1*. In this case, the subpopulation *i + 1* decreases its population size due to the bottleneck. The equations with the binomial distribution were run 100 times. We ran the simulation using R software version 3.5.0^2^.

### Statistical Analysis

Before confirming statistical differences with reference values or between two groups, the Shapiro–Wilk test was conducted to test whether the variables were normally distributed. Moreover, the homogeneity of variance was validated using the *F*-test if the dataset followed a normal distribution. For chlorine sensitivity, both two-sided and lower-tailed one-sample *t*-tests were applied. The two-sided Welch’s *t*-test was also conducted to compare the cell-binding ratio and specific growth rate between the less-sensitive and sensitive populations. Statistical differences in the infectious titer, lag period, and non-synonymous and synonymous diversities were assessed using the Wilcoxon rank-sum test. To confirm the correlation of LRV with non-synonymous and synonymous nucleotide diversities, robust linear regression, which can minimize the effect of outliers when datasets are fitted, was conducted using the scikit-learn library in Python version 3.7 (see footnote 1). Pearson product-moment correlation coefficient and Spearman’s rank correlation coefficient were estimated to find a correlation between LRV and nucleotide diversities and the frequency of VP7 SNPs identified by linear SVM. Except for the robust regression analysis, all statistical tests were performed using R software version 3.5.0.

## Results

### Random Generation of Rhesus Rotavirus Populations Less Sensitive to a Disinfectant

**FIGURE 3:**
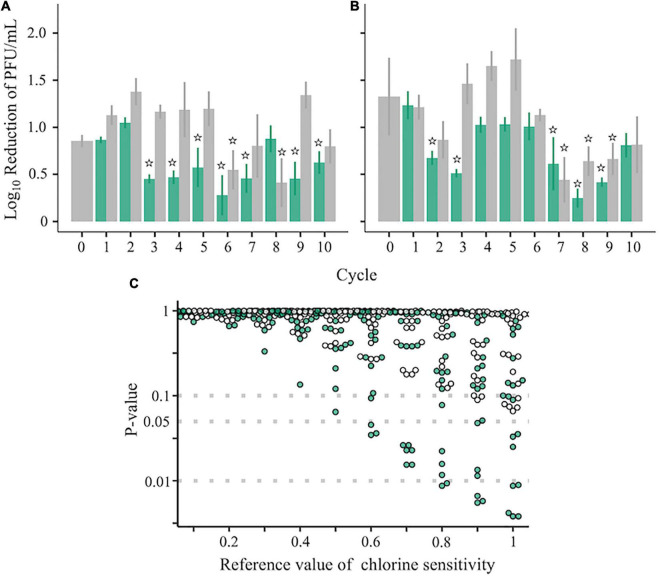
Chlorine sensitivity of the RRV populations. **(A–C)** Chlorine sensitivity of 42 RRV populations in the first **(A)** and second **(B)** groups. Green and gray plots with standard deviation (*N* = 3) are assigned to populations passaged under chlorine disinfection and dilution, respectively. Populations less sensitive to chlorine are marked with stars that indicate no significant differences in the 0.5 log_10_ reduction value (one-sampled *t*-test, *P* > 0.05). **(C)** *P*-values of 42 populations estimated by the one-sided one-sample *t*-test (lower tail) based on the reference chlorine sensitivity ranged from 0.1 to 1.0 (green: chlorine experienced populations; gray: no chlorine experienced populations through serial passages).

Two ancestral RRV populations, which had different disinfection sensitivities and genetic diversities (Figure 1), were serially passaged 10 times to test whether a population less sensitive to chlorine appeared. Before inoculation of the virus into the host cell, the virus samples were inactivated using chlorine (Test) or were merely diluted (Control) (Figure 1). The sensitivity to disinfection randomly changed through the serial passages (Figures 3A,B). To determine which populations were significantly less sensitive to chlorine disinfection, we used the two-sided one-sample *t*-test with a 0.5 LRV, which was 50% of the expected disinfection sensitivity (90% reduction ≒ 1.0 LRV). As a result, the disinfection sensitivity of 17 populations, including 12 Test and 5 Control populations, did not achieve the expected disinfection sensitivity (no significant difference from 0.5 LRV; *P* > 0.05). We defined these 17 populations as “less-sensitive populations A; LA” (sensitive populations A were expressed as SA). To discover populations that were more resistant to chlorine from LA, the mean LRVs of each population were compared with the hypothetical LRVs ranging from 0.1 to 1.0 in the lower-tailed one-sample *t*-test (Figure 3C). When the hypothetical value of the *t*-test was 1.0, the *P*-value of 8 populations was less than 0.05. In contrast, there were no less-sensitive populations when the hypothetical LRVs were less than 0.5 (Figure 3C). When the hypothetical LRV was 0.6, the mean values of the three populations were significantly lower than the hypothetical values (*P* < 0.05); thus, we defined these three populations as “less-sensitive populations B; LB” (sensitive populations were expressed as SB).

### Virological Phenotypes

**FIGURE 4:**
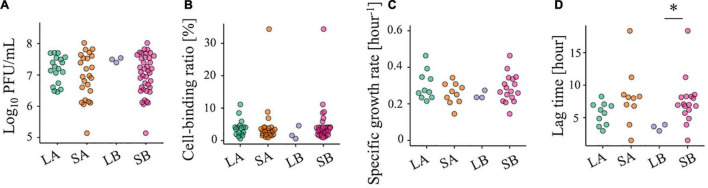
Evaluation of the viral phenotypes of 42 RRV populations. **(A)** Plaque-forming units per milliliter (PFU/ml) of less-sensitive populations (LA and LB) and sensitive populations (SA and SB) (red: Control populations of the first group; blue: Control populations of the second group; green: Test populations of the first group; purple: Test populations of the second group; *N* = 3). **(B)** Cell binding ability of LA, SA, LB, and SB. **(C,D)** Specific growth rate and lag period of the 10 LA, 10 SA, 3 LB, and 10 SB populations. Statistical differences were tested using the Wilcoxon rank-sum test. The asterisk means that *P*-value is less than 0.05.

We also tested some virological phenotypes of each population to explore clues to the cause of chlorine sensitivity reduction. There were no significant differences in infectious titer, specific growth rate, and cell-binding efficiency between the less-sensitive and sensitive populations (Figures 4A–C), whereas a lower *P*-value for the lag time was observed for LB (Figure 4D, two-sided Wilcoxon rank-sum test; *P* = 0.02 for LB vs. SB). However, low values of the lag time were also found in some of the SA and SB populations, indicating that the chlorine sensitivity was not attributed to these virological phenotypes.

### Calling Single Nucleotide Polymorphisms

The genomic diversity of the RRV populations was evaluated using FLDS on the basis of the frequency and variation of SNPs in the entire genomic segments. The average sequencing read coverage per site was 1,632 (*SD* ± 1131) (Supplementary Table 1) and exceeded the reference values recommended in a previous study on the identification of mutations (Jennings et al., 2017). Since all the nucleotide positions with SNPs had high Phred scores (32.2 at minimum, corresponding to <0.1% of sequencing errors) (Supplementary Table 2), these sequence data were used for subsequent analyses. Mutations specific to each population were fixed despite using the same treatments, such as disinfection and dilution (VP7: E256G [Control populations of the second group], L150P [Test populations of the second group], P275L, T281I [Test populations of the first group], and NSP4: K86E [Control populations of the first group]) (Supplementary Figure 1), suggesting that population bottlenecks occurred in this study. We also found that the frequencies of minor SNPs changed at random through serial passages, implying that bottlenecks affected the subpopulation structure composed of minor mutants as well.

### High Non-synonymous Genetic Diversity of the Outer Capsid Protein Gene in Less-Sensitive Populations

**FIGURE 5:**
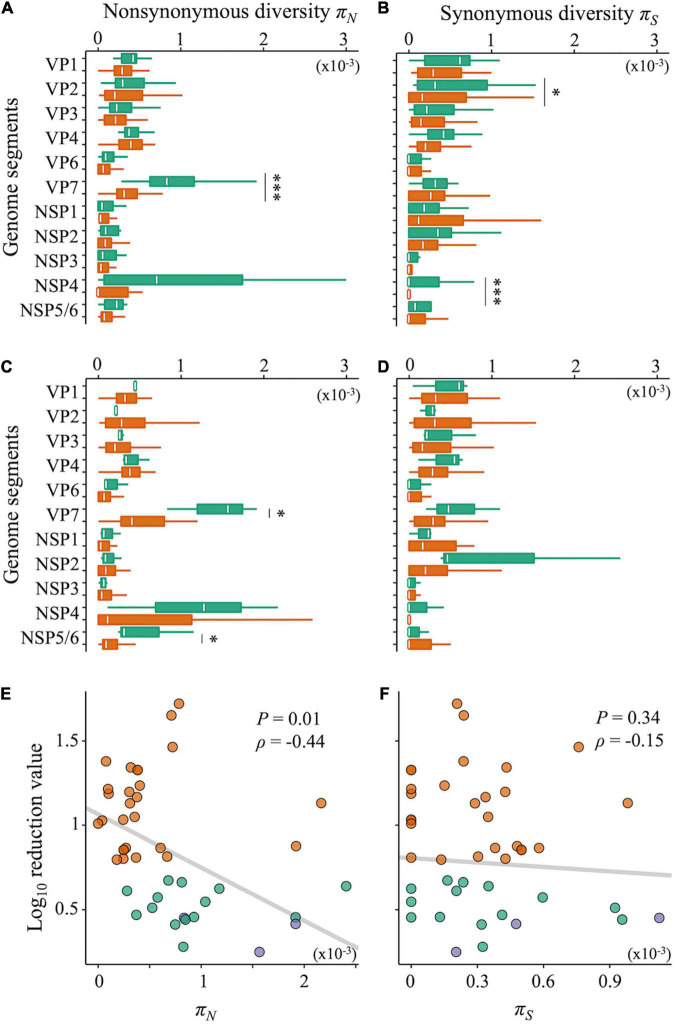
Non-synonymous and synonymous nucleotide diversity. **(A–D)** Nucleotide diversities of each genome segment of RRV were compared between less-sensitive populations A (LA) and sensitive populations A (SA) (**A:** non-synonymous; **B:** synonymous mutations) or less-sensitive populations B (LB) and sensitive populations B (SB) (**C:** non-synonymous; **D:** synonymous mutations). Green bars show the values of LA and LB (*N* = 17 and 3 for LA and LB, respectively), whereas orange ones show those of SA and SB (*N* = 25 and 39 for SA and SB, respectively). Statistical differences between less-sensitive and sensitive populations were confirmed based on Welch’s *t*-test or Wilcoxon rank-sum test (*P < 0.05; ^***^P < 0.001). **(E,F)** Relations of the log_10_ reduction value by chlorine disinfection with non-synonymous **(E)** and synonymous **(F)** diversities of the VP7 genes, respectively (green: LA; blue: LB; orange: SA and SB).

Nucleotide diversity, as an indicator of intrapopulation genetic diversity, denotes the probability that two sequences randomly sampled from a gene pool are different from each other. Both LA and LB exhibited significantly higher nucleotide diversities of the gene coding outer capsid protein VP7 (Supplementary Figures 2A,B, Wilcoxon rank-sum test; *P* < 0.05 for both LA and LB). The nucleotide diversity is composed of non-synonymous (*π_*N*_*) and synonymous (*π_*S*_*) nucleotide diversities. By calculating *π_*N*_* and *π_*S*_*, we can identify which types of nucleotide diversity are more important for disinfection sensitivity. Compared with the sensitive populations, both LA and LB showed significantly higher *π_*N*_* of the VP7 gene (Figures 5A,C, Wilcoxon rank-sum test; *P* < 0.05 for both LA and LB). The *π_*S*_* of the VP1 and NSP4 genes of LA were also higher, whereas those of LB was not different from that in sensitive populations (Figures 5B,D). We found a negative correlation between the disinfection sensitivity and the *π_*N*_* of VP7 (Figure 5E; Pearson’s *r* = –0.37, *P* = 0.01; Spearman’s ρ = –0.44, *P* = 0.003) but no correlation between the disinfection sensitivity and the *π_*S*_* (Figure 5F; Pearson’s *r* = –0.18, *P* = 0.24; Spearman’s ρ = –0.15, *P* = 0.34).

### No Specific Mutations Are Associated With the Chlorine Disinfection Sensitivity

**FIGURE 6:**
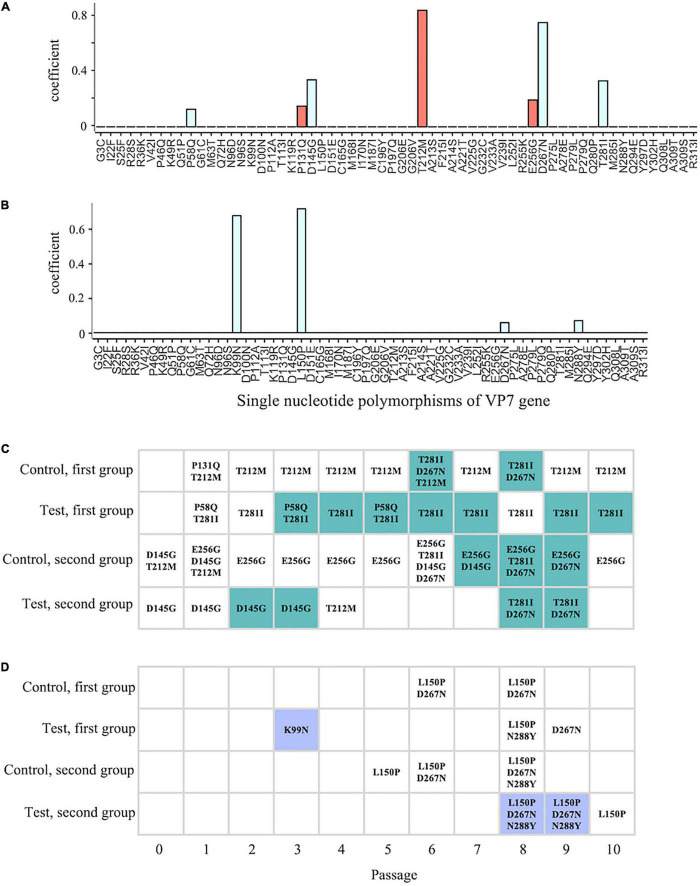
Comparison between the timing of the appearance of the less-sensitive population and SNPs. **(A,B)** Coefficients of individual SNPs for classification into less-sensitive and sensitive populations were estimated using the L1 linear support vector machine (SVM) with SNP frequency [**A,B:** classification into less-sensitive populations A (LA) and B (LB), respectively]. Red and blue bars indicate the positive and negative values, respectively. **(C,D)** The colored cells indicate that a less-sensitive population occurs in the lineage at *i^th^* time of serial passage (**C:** LA; **D:** LB), and the point of time at which the less-sensitive populations appeared was compared with that at which SNPs appeared, demonstrating high contribution to the classification (LA: P58Q, P131Q, T212M, E256G, T281I, D145G, and D267N; LB: K99N, L150P, D267N, and N288Y).

The disinfection sensitivity is likely rooted in the intrapopulation genetic diversity of the VP7 gene (Figure 5). This can be explained by increases in the frequency of a few specific sequences (natural selection) or a wide variety of minor sequences. We, therefore, postulated two working hypotheses to explain how virus populations with high non-synonymous diversity of the VP7 gene acquired lower sensitivity to chlorine. The first hypothesis is based on natural selection, in which several specific mutants enabled the population to resist chlorine disinfection. We applied the L1 linear SVM to identify the VP7 SNPs important for classification into the less-sensitive group. Generally, a classification algorithm works well when a number of variables are available; however, in this study, the performance of the L1 linear SVM was compatible with that of the L2 linear SVM that used all variables for classification (L1 score = 0.67 and 0.91 and L2 score = 0.61 and 0.91 for LA and LB, respectively). Thus, the L1 linear SVM was able to select the SNPs necessary and sufficient for the classification from all VP7 SNPs and showed that seven SNPs (P58Q, P131Q, D145G, T212M, E256G, D267N, and T281I) of LA had non-zero coefficients (Figure 6A). In contrast, the classification into LB needed only four SNPs (K99N, L150P, D267N, and N288Y) (Figure 6B). The shared SNP between LA and LB was only D267N.

Correlation analysis between the extracted SNPs and chlorine sensitivity revealed significant correlation coefficients for D267N (Pearson’s *r* = –0.38, *P* = 0.01; Spearman’s ρ = –0.42, *P* < 0.01) (Supplementary Table 3). However, we noted that not all less-sensitive populations had D267N (6 populations in LA and 2 populations in LB) (Figures 6C,D). K99N was present in low frequency in one of the LB populations (1.88%) and did not remain in the subsequent generations (Figure 6D). As for LA, T281I significantly correlated with chlorine sensitivity (Pearson’s *r* = –0.44, *P* < 0.01; Spearman’s ρ = –0.46, *P* < 0.01). As for the LB result, however, not all less-sensitive populations had T281I. If the disinfection sensitivity is determined by specific mutants, advantageous mutations must persist in even less-sensitive populations, and the low sensitivity must be maintained according to the principle of natural selection. Thus, the reduction in chlorine sensitivity is unlikely to be associated with any specific mutations.

### *In silico* Virus Clusters Increase Diversity and Destabilize Disinfection Sensitivity

An alternative hypothesis was formulated to explain the observation that a wide variety of mutants within a population contributed to the reductions in disinfection sensitivity. We further assumed that mutants in genetically diverse populations had different physical properties in terms of the virion surface (e.g., electrostatic potential and hydrophobicity), which promoted the formation of virus clusters comprising not only major but also minor mutants with an affinity toward each other. Such clusters were assumed to avoid damage to the whole region of capsid protein and/or enable the virion that could not infect alone to replicate *via* coinfection with intact virions within a cluster (i.e., cooperation). Recently, it has been reported that viral phenotypes are modified by positive interactions among variants (i.e., cooperation) (Shirogane et al., 2016). To confirm whether and how virus clusters formed in a genetically diverse population impacted the disinfection sensitivity of the population, we tracked the sizes of the virus subpopulations in the *in silico* viral population under a disinfection-exerted environment by using ordinary differential equations.

**FIGURE 7:**
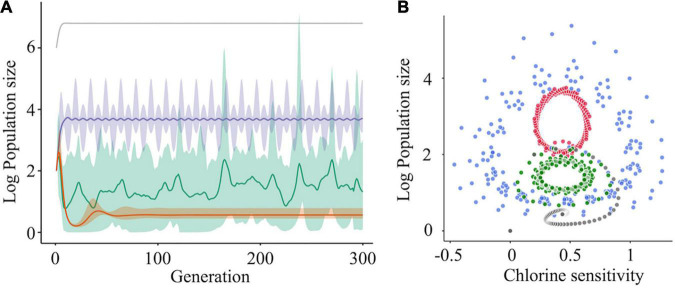
The effect of cooperative interaction between mutants on population diversity and chlorine sensitivity. **(A)** Simulations of the log_10_ population size of subpopulations (one was a major subpopulation [gray] and the others were minor subpopulations) were run under three scenarios for the minor subpopulations: no interaction (orange), cooperation (purple), and cooperation with the bottleneck (green). When cooperation was exerted among the minor subpopulations, the sizes of the cooperative mutants increased with fluctuation and became stable (purple). Random elimination of interaction among mutants (i.e., bottleneck) randomized the genetic diversity within a population (green). The solid lines and shades express the average values and 95% confidence intervals, respectively. **(B)** Solution orbits of the log_10_ population size of a mutant non-interacting (gray) or cooperatively interacting with two (green), four (blue), and six (red) other mutants have been depicted.

The simulation was run for one major subpopulation having a higher fitness *r* (e.g., growth ability) to maintain dominance in a population and 10 subpopulations that did not have cooperative interactions with others. In this case, log_10_ subpopulation sizes were converged to a lower level (approximately 1) (Figure 7A, orange curve). We then assumed that genetically diverse clusters are formed by five different sequences, and we found that the population sizes of the cooperative subpopulations increased with fluctuations and finally reached approximately 4 (Figure 7A, purple curve). This indicates that virus clusters increase the genetic diversity of a population and avoid extinction. However, the simulation results did not reflect the random change in genetic diversity observed in this study (Figures 3, 5). To reproduce the phenomenon, some cooperative subpopulations were stochastically excluded from a population by incorporating artificial bottleneck events into the model. As a result, the intrapopulation genetic diversity randomly changed and was maintained at high levels (Figure 7A, green curve); this explains the observed randomness of the intrapopulation genetic diversity and disinfection sensitivity (Figures 3, 5). These simulation results suggest that the virus clusters maintain high genetic diversity under chlorine disinfection and that a population bottleneck plays a critical role in random changes in the intrapopulation genetic diversity.

The relationship between the population size and chlorine sensitivity of a cooperative subpopulation was confirmed using four-phase plots in which the number of cooperators varied (Figure 7B). Without cooperation, the population size and chlorine sensitivity converged clockwise to a certain point. When other cooperative subpopulations existed, the solution did not converge to a unique point. In the case that there were six other cooperative subpopulations in a population, the population size changed periodically between 0.25 and 0.7 values of disinfection sensitivity (Figure 7B). At times, solutions of seven subpopulations turned inward but immediately returned outward, indicating that cooperative virus clusters prevent the disinfection efficacy from converging to a unique point. If a bottleneck is present in the diverse population, the chlorine sensitivity deviates from the periodic orbit in the manner of stochasticity. In other words, less-sensitive populations emerge at random due to a bottleneck and cooperation. Virus cooperation could influence both the intrapopulation genetic diversity and the disinfection sensitivity.

## Discussion

In this study, RRV populations less-sensitive to chlorine disinfection randomly emerged from serial passages (LA: 11 Test and 6 Control populations; LB: 3 Test populations) (Figure 3). As reduced disinfection sensitivity was not attributed to infectivity, cell binding ability, and specific growth rate (Figures 4A–C), we examined whether disinfection sensitivity was in any way related to the quasispecies structure by estimating the intrapopulation genetic diversity. We observed that the non-synonymous nucleotide diversity of the outer capsid protein gene VP7 was higher in less-sensitive populations than in sensitive ones (Figures 5A,C). A non-synonymous substitution of VP7 may alter the secondary and tertiary structures of this viral capsid protein, resulting in changes in the virion stability and electrostatic potential of viral particles. The disinfection sensitivity was not well explained by specific mutations identified by the L1 linear SVM, which meant that there were no common mutations among the less-sensitive populations (Figures 6C,D). We then hypothesized that the disinfection sensitivity changes were attributable to the existence of cooperative virus clusters within a population. The evolutionary simulation results suggested that the formation of a virus cluster and the bottleneck were associated with the genetic diversity within a population as well as the disinfection sensitivity (Figure 7).

First, one must confirm whether the RRV populations used in this study indeed exist as quasispecies. The two ancestral populations included distinct SNPs and nucleotide diversities (Figure 1 and Supplementary Figure 1). The nucleotide diversities from the start to the end of the serial passages were non-zero values (Figures 1, 5), and all populations had a large number of SNPs (47.0 ± 8.7 per population) (Supplementary Figure 1). In addition, true positive SNPs beyond the sequencing errors were certainly identified due to the high sequencing quality (Supplementary Table 2). The quasispecies nature of the RRV populations was also verified in our previous study; we proved that several haplotypes existed in initial populations in which the same RRV populations were used (Kadoya et al., 2020). Furthermore, the verification of the quasispecies nature was supported by experiments involving serial passages of murine rotavirus (Tsugawa et al., 2014).

The populations less sensitive to chlorine disinfection (LA and LB) were selected from 42 populations according to two scenarios in which different reference values of statistical tests were used. Both LA and LB had higher genetic diversities of the outer capsid protein gene VP7 (Figures 5A,C); thus, the hypothesis that intrapopulation genetic diversity is the main driver of changes in chlorine sensitivity is plausible. The hypothesis is also supported by simulation results in which cooperative virus clusters within a highly diverse population destabilize their disinfection sensitivity (Figure 7). Under the concept of sociovirology (Díaz-Muñoz et al., 2017), variants within a viral population can aid each other and transmit as a cluster. This helps in maintaining the genetic diversity at a high level and modifying the phenotype of a population (Vignuzzi et al., 2006; Combe et al., 2015; Andreu-Moreno and Sanjuán, 2018; Ke et al., 2018). Along with these study findings, these previous studies show that the high genetic diversity likely enhances the probability of survival from various hostile pressures in the environment by generating cooperative interactions among individual mutants. Since our result was obtained from a simulation in which we assumed the formation of cooperative virus clusters, we need to experimentally demonstrate the formation of cooperative clusters in a genetically diverse virus population, for example, by using high-performance particle counters, in further studies.

The random change in the disinfection sensitivity of the RRV populations can be explained by the bottleneck. Our evolutionary simulation results suggested that the cooperatively formed virus clusters destabilized the disinfection sensitivity and that the genetic diversity of the population was high when the clusters remained in a population under a bottleneck (Figure 7). The exclusion of cooperative virus clusters due to a bottleneck decreases the genetic diversity. In this case, cooperation to form virus clusters can no longer be possible in future populations. However, the bottleneck can result in exploring a new sequence space *via* the exclusion of major sequences and allows the hidden rare sequences to get some space (i.e., an increase in the intrapopulation genetic diversity). The strength of the bottleneck determines whether the bottleneck performs its exploration (Kadoya et al., 2020). When minor sequences in a cluster acquire a new sequence space, the genetic diversity increases, while the chlorine sensitivity can remain low. If the clusters fail to transmit, the population may react more to disinfection, and the genetic diversity will decrease. However, the genetic diversity cannot reach zero because the sequence space explored by the mild bottleneck is available to the remaining minor sequences. Such populations with non-zero diversity may generate a virus cluster that is less sensitive to disinfection in future generations.

The transmission mode of a virus cluster assumed in this study is analogous to en-bloc transmission. During en-bloc transmission, genetic diversity is maintained at high levels due to host-to-host transmission as a cluster including multiple virions; moreover, cooperation among mutants can be promoted (Chen et al., 2015; Combe et al., 2015; Sanjuán, 2017). Virologists have claimed that cooperation explains the pathogenesis or infectivity at the virus population level (Vignuzzi et al., 2006; Shirogane et al., 2019). For example, the pathogenic potential of poliovirus was restored after exposure to a chemical mutagen that increased its genetic diversity; the cooperative interaction within the population certainly existed (Vignuzzi et al., 2006). This study proposes that cooperative behaviors may contribute to the change in disinfection sensitivity and provides novel insights based on the fact that cooperation can enable a population to survive adverse pressures existing not only within the host but also in natural environments. Several points need to be unraveled to more elaborately explaining the mechanism underlying disinfection sensitivity changes. The formation of virus clusters in a highly diverse population needs to be experimentally proved by using nanoparticle tracking systems in the future study. The causes underlying the formation of virus clusters may be identified by estimating the effect of amino acid replacements on the physical properties of the virions.

There are several limitations to this study. First, the initial virus concentration for the serial passage experiment was approximately 10^5^ PFU/ml, and in further study, we need to confirm how different initial concentrations affect the genetic diversity and disinfection sensitivity of a population. Second, it is necessary to confirm whether the mechanism of disinfection sensitivity change, which we proposed, in this study, is common even under the other disinfection intensities. It is also necessary to confirm whether wild-type rotavirus populations, found in samples from human feces or wastewater, are really less sensitive to disinfection when the intrapopulation genetic diversity is high. Alternatively, we may be able to assess whether a population, the genetic diversity of which is artificially enhanced like that described in Vignuzzi et al. (2006), shows less sensitivity to chlorine disinfection. The plaque-forming capability of virus clusters also needs to be evaluated. Since virions forming a cluster may be able to infect host cells adjacent to each other or coinfect identical cells, it is speculated that plaques formed by each virion in a cluster are easily overlapped and sometimes are difficult to be separately counted. This means that the infectious titer of less-sensitive populations can be smaller than that of sensitive ones owing to the presence of clusters when both populations include the same number of virions. However, we observed that less-sensitive populations gave a higher number of plaques after chlorine disinfection than sensitive ones, which must not be caused by the cluster formation. To verify this hypothetical role of clusters in less-sensitive populations, it is necessary to compare the plaque-forming capabilities of a cluster and dispersed virions in the further study.

Despite the introduction of hygiene practices, RNA viruses have caused severe outbreaks globally (de Graaf et al., 2015; Burke et al., 2018; Church et al., 2019). In addition, previous studies have reported that the genetic diversities of some waterborne viruses, which are associated with current outbreaks, have increased (Hassel et al., 2017; Sabrià et al., 2018; Lema et al., 2019). In this study, we demonstrated that genetically diverse populations had low sensitivity to chlorine. Disinfection is an important practice that can help decrease the RNA virus infection burden; however, an increase in the intrapopulation genetic diversity of RNA virus populations may decrease the disinfection efficiency. As indicated herein (Figures 3, 7), if disinfection acts as one of the bottlenecks in the environment, waterborne viruses will not become resistant to disinfection, and we will be able to sufficiently inactivate those using appropriate disinfection practices. However, disinfection will not work well as long as cooperative virus clusters exist. Disinfection strategies need to be developed considering the effect of cooperative virus clusters on disinfection efficiency at wastewater treatment plants and medical facilities. Although more studies that the cooperative clusters exist in a genetically diverse population are required, the novel hypothesis we derived in this study can be valuable to verify whether or not it is one of the styles of RNA virus evolution in the further study. Overall, ensuring efficacious prevention measures in terms of disinfection practices requires monitoring the genetic diversity of the virus as well as its concentration in water and on environmental surfaces.

## Data Availability Statement

The datasets presented in this study can be found in online repositories. The names of the repository/repositories and accession number(s) can be found below: https://www.ncbi.nlm.nih.gov/genbank/, DRA006847; https://www.ncbi.nlm.nih.gov/genbank/, DRA009378.

## Author Contributions

S-SK, S-IU, TNu, SO, ONa, and DS designed the research. S-SK, performed the experiments related to the evaluation of the rotavirus phenotypes, the evolutionary simulation, and statistical analyses. ONa and TNa designed the experimental system using rotavirus and prepared the initial rotavirus populations. S-IU, MH, and TNu developed and performed the NGS processing. MK, SO, and DS developed the approach for phenotypic evaluation and conducted disinfection experiments. S-SK, S-IU, and YT conducted the population genetic analyses. ONi participated in data and statistical analyses. DS coordinated and supervised this study. S-SK, S-IU, TNu, and DS drafted the manuscript. All the authors have read and agreed to this version of the manuscript.

## Conflict of Interest

The authors declare that the research was conducted in the absence of any commercial or financial relationships that could be construed as a potential conflict of interest.

## Publisher’s Note

All claims expressed in this article are solely those of the authors and do not necessarily represent those of their affiliated organizations, or those of the publisher, the editors and the reviewers. Any product that may be evaluated in this article, or claim that may be made by its manufacturer, is not guaranteed or endorsed by the publisher.

## Footnotes

1
https://www.python.org/downloads/release/python-372/

2
https://www.r-project.org/
