# Supplementary material for: The Intrapopulation Genetic Diversity of RNA Virus May Influence the Sensitivity of Chlorine Disinfection
Source: Front Microbiol. 2022 May 20;13:839513. doi: 10.3389/fmicb.2022.839513 (PMC9163991; doi:10.3389/fmicb.2022.839513)
Supplement: Supplementary file 1 [file Data_Sheet_1.docx]

S**upplementary Table. 1 Average sequence coverage of rhesus rotavirus populations**

|  | cycle 0 | cycle 1 | cycle 2 | cycle 3 | cycle 4 | cycle 5 | cycle 6 | cycle 7 | cycle 8 | cycle 9 | cycle 10 |
| --- | --- | --- | --- | --- | --- | --- | --- | --- | --- | --- | --- |
| 1st test | 2706 | 2520 | 3293 | 3015 | 3669 | 3203 | 375 | 224 | 684 | 687 | 643 |
| 1st control | - | 2486 | 4088 | 1775 | 1718 | 3268 | 474 | 1202 | 2023 | 587 | 2353 |
| 2nd test | 1004 | 390 | 525 | 890 | 1081 | 1676 | 1378 | 212 | 1054 | 687 | 301 |
| 2nd control | - | 2517 | 3019 | 3009 | 1176 | 2978 | 2978 | 245 | 949 | 358 | 862 |

**Supplementary Table.2 Average sequencing read quality of rhesus rotavirus populations**

|  | cycle 0 | cycle 1 | cycle 2 | cycle 3 | cycle 4 | cycle 5 | cycle 6 | cycle 7 | cycle 8 | cycle 9 | cycle 10 |
| --- | --- | --- | --- | --- | --- | --- | --- | --- | --- | --- | --- |
| 1st test | 37.03 | 36.71 | 36.77 | 36.94 | 36.98 | 36.81 | 35.07 | 34.80 | 33.97 | 35.24 | 35.17 |
| 1st control | - | 36.62 | 36.65 | 36.65 | 36.82 | 36.89 | 33.54 | 32.21 | 33.90 | 33.99 | 33.46 |
| 2nd test | 36.96 | 37.00 | 37.27 | 37.27 | 37.01 | 36.91 | 34.73 | 35.25 | 33.96 | 33.97 | 34.37 |
| 2nd control | - | 36.86 | 37.11 | 37.11 | 36.99 | 37.14 | 33.72 | 35.06 | 35.26 | 35.75 | 35.66 |

**Supplementary Table. 3 Correlation between log_10_ reduction value and SNPs identified by linear support vector machine**

|  | P58E | K99N | P131E | P145E | L150P | T212M | E256G | D267N | T281I | N288Y |
| --- | --- | --- | --- | --- | --- | --- | --- | --- | --- | --- |
| Pearson's *r* | -0.20 | -0.18 | 0.10 | -0.02 | -0.22 | 0.25 | 0.23 | -0.38 | -0.44 | -0.26 |
| P-value | 0.18 | 0.25 | 0.50 | 0.93 | 0.16 | 0.11 | 0.13 | 0.01 | <0.01 | 0.08 |
| Spearman's *ρ* | -0.19 | -0.20 | 0.10 | 0.09 | -0.24 | 0.27 | 0.20 | -0.42 | -0.46 | -0.26 |
| P-value | 0.23 | 0.20 | 0.51 | 0.57 | 0.11 | 0.08 | 0.18 | <0.01 | <0.01 | 0.08 |


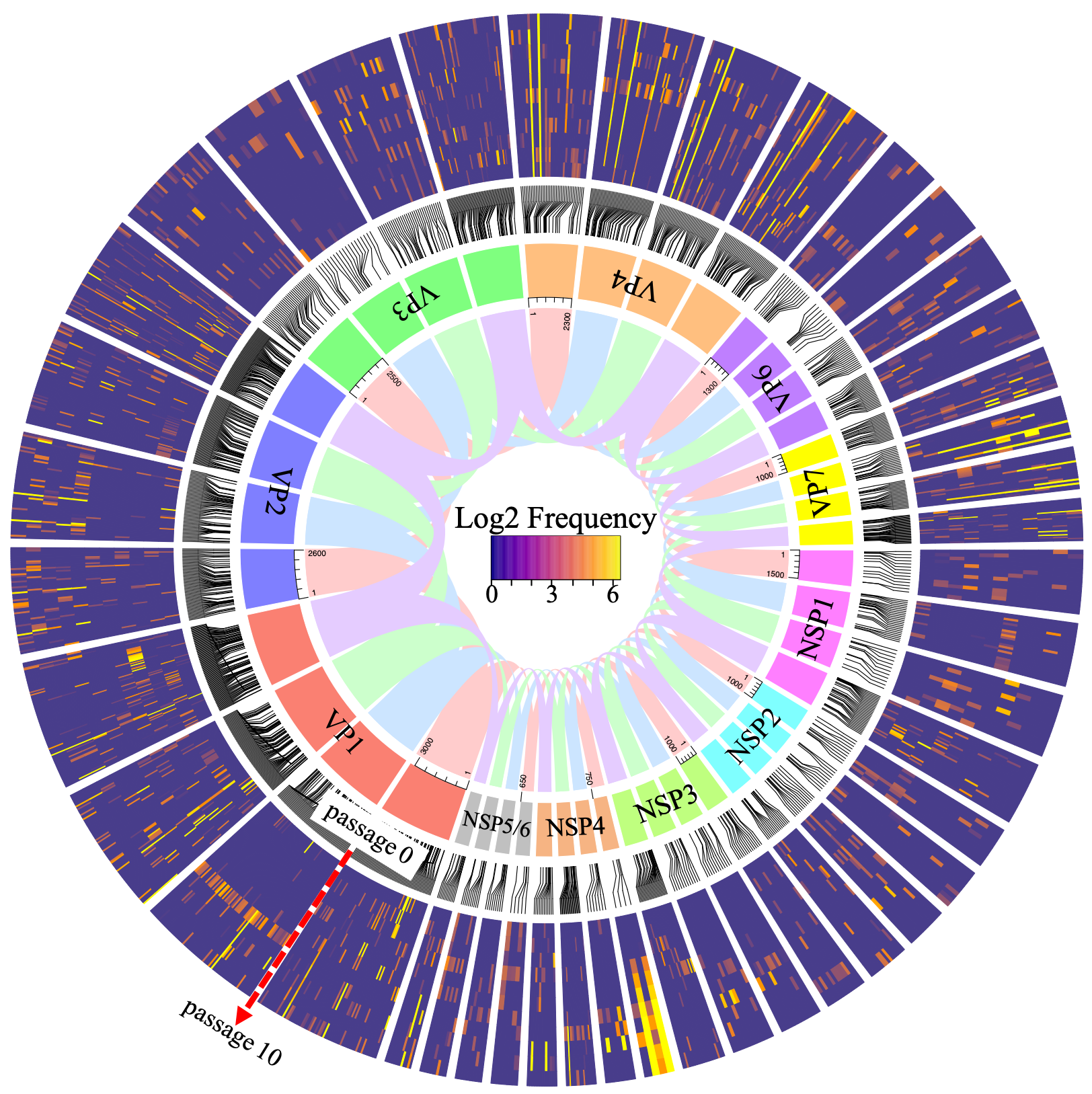


**Supplementary Fig. 1 Transition of single nucleotide polymorphism (SNPs) frequency**

Frequency of each SNP was transformed to the base 2 logarithm corresponding to the tint (high frequency: yellow, low frequency: navy) and the changes of SNPs frequency during serial passages were seen from the inside to the outside of a circos plot (red arrow). A heatmap cell was arranged clockwise from VP1 to NSP5/6 showing the location of SNPs on genome segments. Colored ribbons by red, green, blue and purple indicates first control, first test, second test and second control populations, respectively.


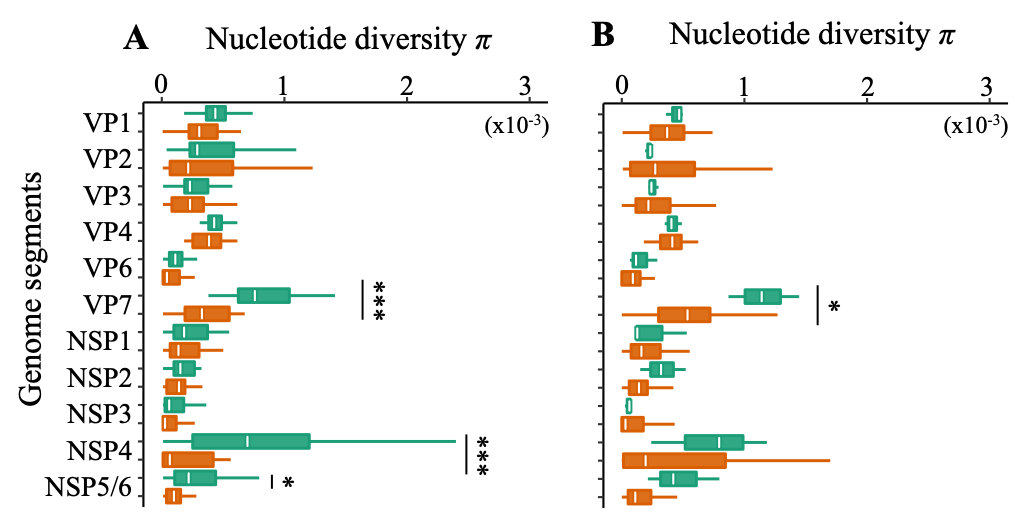


**Supplementary Fig. 2** **Nucleotide diversity**

Nucleotide diversity for each genome segment (green: less-sensitive, orange: sensitive populations) of less-sensitive populations A (**A**) and B (**B**). Statistical differences between less-sensitive and sensitive populations were confirmed based on Wilcoxon's rank-sum test (**P* < 0.05, ****P* < 0.001).
